# Seasonal ambient air pollution correlates strongly with spontaneous abortion in Mongolia

**DOI:** 10.1186/1471-2393-14-146

**Published:** 2014-04-23

**Authors:** Davaasambuu Enkhmaa, Nicole Warburton, Badrakh Javzandulam, Jadambajav Uyanga, Yarinpil Khishigsuren, Sereeter Lodoysamba, Shonkuuz Enkhtur, David Warburton

**Affiliations:** 1 National Center for Maternal and Child Health, Khuvisgalchdin Street, Bayangol District, Ulaanbaatar, Mongolia; 2 Mills College, 5000 MacArthur Boulevard, Oakland, CA 94613, USA; 3 The National University of Mongolia, Ikh surguuliin gudamj-1, Baga toiruu, Sukhbaatar District, Ulaanbaatar, Mongolia; 4 The Lifespan Environmental Pollution Global Impact Center at The Saban Research Institute, Children’s Hospital of Los Angeles, 4650 Sunset Boulevard MS35, Los Angeles, CA 90027, USA

**Keywords:** Air pollution, Fetal death, Mongolia, Seasonal variation, Spontaneous abortion

## Abstract

**Background:**

Air pollution is a major health challenge worldwide and has previously been strongly associated with adverse reproductive health. This study aimed to examine the association between spontaneous abortion and seasonal variation of air pollutants in Ulaanbaatar, Mongolia.

**Methods:**

Monthly average O_3_, SO_2_, NO_2_, CO, PM_10_ and PM_2.5_ levels were measured at Mongolian Government Air Quality Monitoring stations. The medical records of 1219 women admitted to the hospital due to spontaneous abortion between 2009–2011 were examined retrospectively. Fetal deaths per calendar month from January-December, 2011 were counted and correlated with mean monthly levels of various air pollutants by means of regression analysis.

**Results:**

Regression of ambient pollutants against fetal death as a dose–response toxicity curve revealed very strong dose–response correlations for SO_2_ r > 0.9 (p < 0.001) while similarly strongly significant correlation coefficients were found for NO_2_ (r > 0.8), CO (r > 0.9), PM_10_ (r > 0.9) and PM_2.5_ (r > 0.8), (p < 0.001), indicating a strong correlation between air pollution and decreased fetal wellbeing.

**Conclusion:**

The present study identified alarmingly strong statistical correlations between ambient air pollutants and spontaneous abortion. Further studies need to be done to examine possible correlations between personal exposure to air pollutants and pregnancy loss.

## Background

Over the last decades, numerous studies have confirmed a positive relation between air pollution and morbidity and mortality [1-4]. Air pollution has previously been strongly associated with adverse reproductive health. Several studies have examined the effects of air pollution on pregnancy, providing evidence that exposure to ambient air pollutants is associated with poor birth outcome, such as low birth weight [5-7], small for gestational age [8-10], preterm birth [11-14], congenital malformations [15-17] and pregnancy complications such as preeclampsia [18]. In contrast, limited data are available on toxic effects of air pollution on pregnancy loss. Previous studies have shown that environmental tobacco smoke is associated with an increased risk of spontaneous abortion [19-21]. One retrospective epidemiological study provided evidence for an association between brief exposure to high levels of ambient particulate matter during the preconceptional period and early pregnancy loss and found a 2.6-fold increased risk of spontaneous miscarriage [22]. More recent investigations in China [23] and Iran [24] also reported an increased risk of fetal loss in early pregnancy during exposure to high levels of air pollutants. However, no report has yet addressed associations between ambient air pollution and pregnancy loss in Mongolia.

Ulaanbaatar (UB), Mongolia is one of the most air polluted capital cities in the world, with ambient sulfide dioxide (SO_2)_ and particulate matter (PM)_10_ and PM_2.5_ levels >23 times World Health Organization (WHO) standards in winter [25]. Despite its extraordinarily high air pollution concentrations, UB has received very little research attention, where 623 deaths attributable to air pollution representing 4.0% of the annual deaths for the entire country [26]. Yet its air quality becomes relatively clean in summer. This is because UB is also the coldest capital city in the world, so that air pollution in wintertime is largely caused by coal burning in Ger stoves for domestic heating [25,27-29]. Moreover, a recent World Bank report relates that these noxious levels of winter air pollution are associated with adverse health effects including cardiovascular events as well as pulmonary diseases that are estimated to cost as much as 19% of UB’s GDP [27].

Herein, we examined the association between spontaneous abortion and seasonal variation of air pollutants measured near the National Center for Maternal and Child Health (NCMCH), which provides the majority of public obstetric and gynecological services in UB.

## Methods

The medical records of 1219 women residing near the Bayangol district and admitted to the hospital due to spontaneous abortion between 2009–2011 were de-identified and examined retrospectively. The information collected included maternal age, history of previous pregnancy, while the presence of other serious medical or systemic conditions were excluded. Spontaneous abortion or fetal death was defined as absence of fetal heartbeat detected by ultrasound prior to 20 weeks of gestational age. Pregnancies ending after that period of gestation were considered to be stillbirths and were excluded.

### Pollution monitoring

Monthly average ozone (O_3_), SO_2_, nitrogen dioxide (NO_2)_, carbon monoxide (CO), PM_10_ and PM_2.5_ levels were measured at City Monitoring Agency’s stations located in the Bayangol district near the NCMCH as indices of monthly average ambient air pollution in that district of UB. Air pollutant levels above the reference levels defined by WHO [30] were considered to be potentially harmful.

### Statistical methods

Spontaneous abortions that occurred from 2009 to 2011 were recorded and expressed as a percentage of all gynecological admissions, as well as analyzed by maternal age. Fetal deaths per calendar month from January-December, 2011 were counted and correlated with mean monthly levels of various air pollutants by means of regression analysis using Excel. Results are expressed as r coefficients with statistical significance accepted with p < 0.05.

### Ethical considerations

Approval for data collection was obtained from the Ethical Review Board at NCMCH and did not require consent to be sought from participants. Personal information was not collected (including the names of mothers or babies, birth dates, addresses, and phone numbers). No further ethics approval was required for the analyses reported here.

## Results

**Figure 1:**
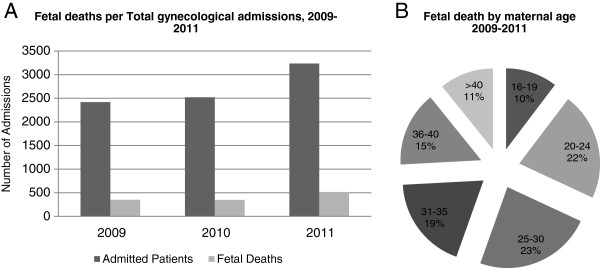
**Fetal deaths in 2009-2011. (A)** per total gynecological admissions. **(B)** by maternal age.

Spontaneous abortion as a proportion of gynecological admissions at NCMCH were consistent over the 3 year period 2009–2011, ranging between 14 and 16% of gynecological admissions per annum (Figure 1A).

Fetal death was distributed evenly across maternal age, reflecting the age distribution of child bearing in the general population (Figure 1B).

In 2011, NCMCH delivered 10,208 babies, while spontaneous abortions totaled 517 (52:1000 live births). However, spontaneous abortion incidence per calendar month ranged over more than 3.6-fold from 20 (23:1000) in May to 75 (73:1000) in December, 2011, revealing a striking seasonal pattern of variation.

Monthly average ambient levels of air pollutants including O_3_, CO, NO_2_, SO_2_, PM_10_ and PM_2.5_ versus WHO air quality guidelines are shown in Table 1. Air pollutant emission was relatively much lower during summer time (June-August) but increased strikingly during the winter months (November-February).

**Table 1:** **Seasonal variation of ambient air pollutants in 2011 versus WHO Air Quality Guidelines (mg/m**^**3**^**)**

**Calendar months**	**O**_**3**_	**O**_**3**_	**SO**_**2**_	**SO**_**2**_	**PM**_**10**_	**PM**_**10**_	**РМ**_**2,5**_	**PM**_**2,5**_	**CO**	**CO**	**NO**_**2**_	**NO**
	**Ambient**	**WHO**	**Ambient**	**WHO**	**Ambient**	**WHO**	**Ambient**	**WHO**	**Ambient**	**WHO**	**Ambient**	**WHO**
1	0.08	0.1	0.08	0.02	0.23	0.05	0.19	0.025	2.72	10.0	0.06	0.04
2	0.04	0.1	0.10	0.02	0.11	0.05	0.11	0.025	2.29	10.0	0.07	0.04
3	0.06	0.1	0.07	0.02	0.14	0.05	0.07	0.025	1.69	10.0	0.05	0.04
4	0.08	0.1	0.05	0.02	0.15	0.05	0.05	0.025	1.08	10.0	0.01	0.04
5	0.07	0.1	0.04	0.02	0.13	0.05	0.04	0.025	0.9	10.0	0.03	0.04
6	0.06	0.1	0.04	0.02	0.08	0.05	0.03	0.025	0.95	10.0	0.02	0.04
7	0.05	0.1	0.04	0.02	0.07	0.05	0.02	0.025	0.77	10.0	0.01	0.04
8	0.06	0.1	0.04	0.02	0.06	0.05	0.02	0.025	0.9	10.0	0.01	0.04
9	0.04	0.1	0.05	0.02	0.10	0.05	0.04	0.025	1.09	10.0	0.01	0.04
10	0.04	0.1	0.06	0.02	0.12	0.05	0.06	0.025	1.53	10.0	0.02	0.04
11	0.04	0.1	0.06	0.02	0.12	0.05	0.09	0.025	1.83	10.0	0.04	0.04
12	0.06	0.1	0.11	0.02	0.15	0.05	0.15	0.025	2.33	10.0	0.05	0.04
**Average**	**0.06**	**0.1**	**0.06**	**0.02**	**0.12**	**0.05**	**0.07**	**0.025**	**1.51**	**10.0**	**0.11**	**0.04**

**Figure 2:**
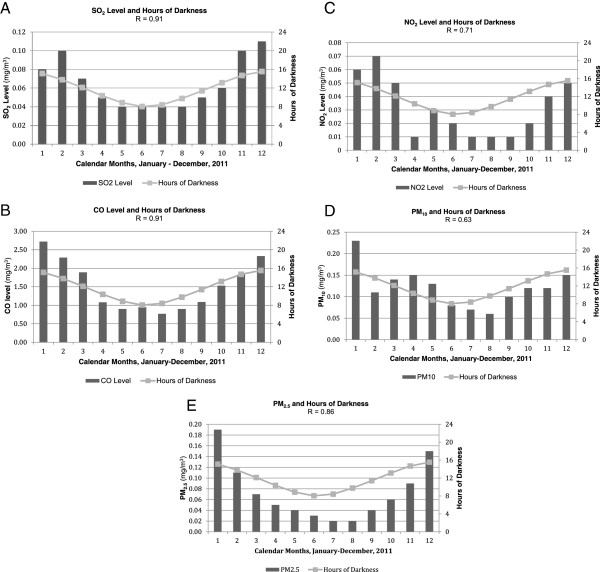
**Calendar monthly averages of ambient air pollutant levels correlated with hours of darkness. (A)** SO_2_. **(B)** CO. **(C)** NO_2_. **(D)** PM_10._**(E)** PM_2.5._ Monthly average air pollutant levels are shown as black bars; seasonal hours of darkness during the annual solar cycle of 2011 are shown as grey dots and a connecting line.

Thus, there was a striking seasonal variation in these mean pollutant levels, which correlated closely with advancing and receding hours of darkness over the annual solar cycle (Figure 2A,B,C,D,E).

**Figure 3:**
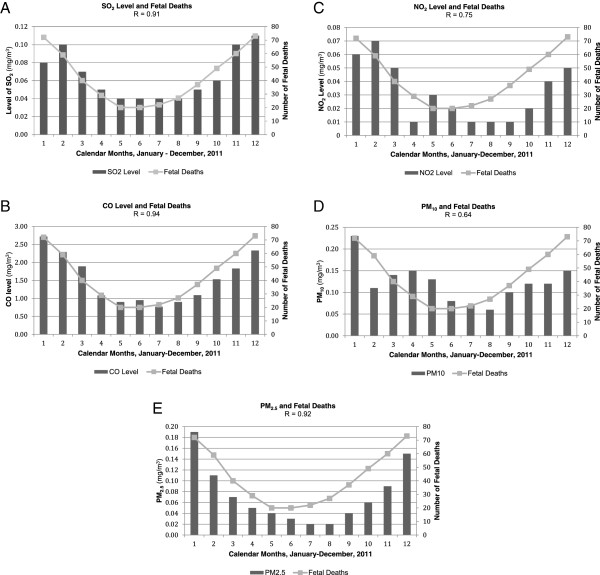
**Calendar monthly averages of ambient air pollutant levels correlated with fetal death. (A)** SO_2._**(B)** CO. **(C)** NO_2._**(D)** PM_10._**(E)** PM_2.5._ Monthly average air pollutant levels are shown as black bars; monthly number of fetal deaths at the National Center for Maternal and Child Health in Ulaanbaatar, Mongolia in 2011 shown as grey dots and a connecting line.

Regression of ambient pollutants against fetal death as a dose–response toxicity curve revealed very strong dose–response correlations for SO_2_ r > 0.9, p < 0.001, while similar strongly significant correlation coefficients were found for NO_2_ (r > 0.8), CO (r > 0.9), PM_10_ (r > 0.9) and PM_2.5_ (r > 0.8), (p < 0.001), indicating an alarming, strongly toxic dose–response correlation between air pollution and fetal death due to spontaneous abortion (Figure 3A,B,C,D,E).

## Discussion

The present study has identified alarmingly strong statistical correlations between seasonal ambient air pollutants and pregnancy loss. Previous studies in China have examined the association between spontaneous abortion before 26 weeks of gestation, and found an increased rate in areas with an elevated mean annual level of hydrogen sulfide (>4 μg/m^3^) [31]. A time-series study in Brazil [32] likewise found a strong association between stillbirth and both NO_2_ as an individual pollutant and an index that combined NO_2_, CO, and SO_2_. Spontaneous abortion has also been associated with environmental tobacco smoke, which contains many of the same chemical pollutants as traffic exhaust [19,20]. An association between ambient CO and both carboxyhemoglobin and nucleated red blood cells, signs of fetal hypoxia, has been previously reported in the venous cord blood of newborns [33]. These levels of CO may interfere with tissue oxygenation levels in the fetus, possibly leading to fetal death, as has been demonstrated in animal models [34]. A recent retrospective case control study in Tehran demonstrated that CO was more toxic with respect to spontaneous abortion than other pollutants, while no significant effect was identified for SO_2_[24]. In contrast, other studies have concluded that exposure to high levels of SO_2_ and total suspended particulate (TSP) but not PM_10_ and NO_2_ during the first month of pregnancy was associated with increased risk of fetal loss [23]. In our study, SO_2_ and CO were more strongly correlated with spontaneous abortion compared to other pollutants. Methodological differences, different geographic and environmental locations affecting factors such as temperature and humidity, as well as different levels of industrialization might explain the variation between these somewhat different results. Although the studies discussed above reported different results in relation to correlation of specific air pollutants with pregnancy loss, all of them have shown that air pollutants have a strong correlation with spontaneous abortion.

Limitations of our study include its retrospective design and the limited nature of pollution observations with respect to peak intradiurnal variation and geographical distribution within UB [28,29]. We did not have available meteorological information on humidity and temperature, but nevertheless we showed that change of air pollutants levels increases in relation to the duration of the hours of darkness. Moreover, because of the retrospective design we were limited in terms of ascertainment of additional biological factors related to spontaneous abortion or fetal death that could be potential confounding factors that might have biased the results.

The Ministry of the Environment and Green Development of the Government of Mongolia, in collaboration with the Millennium Challenge Project and World Bank has made major policy strides in recent years in curbing air pollution, reportedly by as much as 30% in 2013 versus 2011 in some central areas of UB, chiefly by the distribution of up to 120,000 more efficient Ger heating stoves, plus Ger clearance programs. The term Ger refers to the traditional round felt tent used as a portable residence by nomadic Mongolian people. Yet, the disturbingly strong correlation between air pollution indices and fetal death we report herein suggests that much more needs to be done to further ameliorate the toxic effects of air pollution on the human unborn.

As has been noted above, the majority of air pollution in UB occurs in winter and is due to coal burning for domestic heating in Gers and wooden houses within the Ger districts, to ameliorate the extreme cold [25,27-29]. Marked diurnal variation in PM_10_ levels also have been noted previously and have been correlated with stove lighting in the morning and evening, as well as stoking during the early part of the night. We and our colleagues in government and academia in Mongolia are expending concerted efforts to improve stove efficiency and to educate the public about correct and more efficient lighting and stoking procedures to supplement stove replacement efforts. Interestingly, our unpublished observations show that diurnal PM_10_ levels drop significantly during the afternoon and early morning. Moreover, inter-diurnal variation in peak PM_10_ exposure is quite marked during the winter months and this is thought to be inversely related not only to atmospheric temperature but also to wind speed. Additionally, ambient PM_10_ exposure is known to be much higher within Ger districts than within built up apartment neighborhoods, while NO_2_ exposure is highest near major traffic thoroughfares [25,27]. This raises the critical question of whether the toxic dose–response relationship between a panel of pollutants including CO, NO_2_, SO_2_, PM_2.5_ and PM_10_ and fetal death noted here in UB as a whole, is due to average or peak levels of exposure to one or more pollutants or a combination of these adverse conditions. Presently personal exposure monitoring is beginning to be carried out in selected representative populations, including pregnant women, to attempt to answer some aspects of these questions about the dose-toxicity response characteristics of ambient pollution.

## Conclusion

Arguably and alarmingly our data suggest that the strong correlation we have identified between ambient levels of air pollutants and spontaneous abortion in UB may also obtain elsewhere in the world, where similarly deleterious levels of air pollution exist. We speculate that up to 5-fold further reduction in air pollutants in winter will be needed to reduce fetal death rates 4-fold back to levels found in summer in UB. Moreover, even in summer the level of spontaneous abortion is unacceptably high. We plan to continue evaluating personal exposure to air pollutants to determine further deleterious effects on health, including adverse pregnancy outcome.

## Abbreviations

NCMCH: National Center for Maternal and Child Health; PM: Particulate matter; UB: Ulaanbaatar; WHO: World Health Organization.

## Competing interests

The authors declare that they have no competing interests.

## Author’s contributions

DE designed the study, analyzed data and co-wrote the manuscript. NW analyzed data and prepared tables and figures. BJ, JU, YK collected and tabulated data with preliminary data analysis. SL, SE oversaw pollution data collection and quality control. DW oversaw the project and co-wrote the manuscript. All authors approved the final manuscript.

## Pre-publication history

The pre-publication history for this paper can be accessed here:

http://www.biomedcentral.com/1471-2393/14/146/prepub
